# Peptide-Functionalized Iron Oxide Nanoparticles for Cancer Therapy: Targeting Strategies, Mechanisms, and Translational Opportunities

**DOI:** 10.3390/molecules31020236

**Published:** 2026-01-10

**Authors:** Andrey N. Kuskov, Lydia-Nefeli Thrapsanioti, Ekaterina Kukovyakina, Anne Yagolovich, Elizaveta Vlaskina, Petros Tzanakakis, Aikaterini Berdiaki, Dragana Nikitovic

**Affiliations:** 1 Department of Chemical-Pharmaceutical and Cosmetic Products Technology, D. Mendeleev University of Chemical Technology of Russia, 125047 Moscow, Russia; kuskov.a.n@muctr.ru (A.N.K.); kukoviakina.e.v@muctr.ru (E.K.); vlaskina.e.r@muctr.ru (E.V.); 2 Department of Histology-Embryology, Medical School, University of Crete, 70013 Heraklion, Greece; lydia.thrapsanioti@gmail.com (L.-N.T.); petzanakak@gmail.com (P.T.); berdiaki@uoc.gr (A.B.); 3 Faculty of Biology, Lomonosov Moscow State University, 119234 Moscow, Russia; anneyagolovich@gmail.com

**Keywords:** therapeutic peptides, iron oxide nanoparticles (IONPs), peptide–IONP hybrids, targeted cancer nanomedicine, peptide–nanoparticle conjugates, tumor microenvironment modulation, tumor penetration peptides, AI-assisted peptide design, dual-function nanotherapeutics

## Abstract

Therapeutic peptides have emerged as promising tools in oncology due to their high specificity, favorable safety profile, and capacity to target molecular hallmarks of cancer. Their clinical translation, however, remains limited by poor stability, rapid proteolytic degradation, and inefficient biodistribution. Iron oxide nanoparticles (IONPs) offer a compelling solution to these challenges. Owing to their biocompatibility, magnetic properties, and ability to serve as both drug carriers and imaging agents, IONPs have become a versatile platform for precision nanomedicine. The integration of peptides with IONPs has generated a new class of hybrid systems that combine the biological accuracy of peptide ligands with the multifunctionality of magnetic nanomaterials. Peptide functionalization enables selective tumor targeting and deeper tissue penetration, while the IONP core supports controlled delivery, MRI-based tracking, and activation of therapeutic mechanisms such as magnetic hyperthermia. These hybrids also influence the tumor microenvironment (TME), facilitating stromal remodeling and improved drug accessibility. Importantly, the iron-driven redox chemistry inherent to IONPs can trigger regulated cell death pathways, including ferroptosis and autophagy, inhibiting opportunities to overcome resistance in aggressive or refractory tumors. As advances in peptide engineering, nanotechnology, and artificial intelligence accelerate design and optimization, peptide–IONP conjugates are poised for translational progress. Their combined targeting precision, imaging capability, and therapeutic versatility position them as promising candidates for next-generation cancer theranostics.

## 1. Introduction

The treatment of cancer, one of the leading causes of death worldwide, is still a challenge due to the complexity of the mechanisms involved in its development. Cancer progression mechanisms involve cell functions such as proliferation, invasion, adhesion, migration, metastasis and apoptosis [1,2]. To date, the primary treatment methodologies for cancer include chemotherapy, radiotherapy, surgery, and immunotherapy, or a combination of these [3,4]. The disadvantages of the existing treatments include deleterious side effects on healthy tissue and an increased incidence of drug resistance [5,6,7]. One of the new therapeutic tools scientists are investigating to improve cancer therapy is anticancer peptides (APCs).

ACPs are short bioactive peptides (5 to 50 amino acids) acting, similarly to antimicrobial peptides, against cancer cell action [7,8,9]. The latter gives them the advantage to separate normal cells from infected cancer cells [5]. Additional advantages of APCs as therapeutic tools are low molecular mass, simple structure, easy absorption, rapid synthesis, and a low occurrence of drug resistance [10]. ACPs are described as mediators of modulation, including membrane disruption, apoptosis, necrosis, angiogenesis, differentiation, and immunomodulation [7,11].

However, despite these advantages, the clinical translation of free anticancer peptides remains limited by their unfavorable pharmacokinetic profiles and instability in biological environments [10].

While other targeting ligands (e.g., folic acid, antibodies, aptamers) have been used with IONPs, peptides offer unique advantages: high target specificity, modular synthesis, ease of chemical conjugation, lower immunogenicity, and the ability to encode multiple functions (targeting, penetration, therapeutic action) in a single sequence. Moreover, peptides can be engineered to respond to tumor-specific enzymes or pH, enabling triggered drug release. These attributes make peptides particularly suitable for constructing smart, multifunctional IONP platforms [7,8,9].

Limitations of APCs include their short half-lives and susceptibility to proteolysis, which reduce their bioavailability and limit their systemic delivery capacity. In addition, APCs can induce immune responses after treatment and cause toxic effects, such as hemolysis [10]. Nevertheless, when synthesizing APCs, modifications can be applied to increase their advantages and improve their therapeutic potential [4,12]. These modifications include replacing natural amino acids with non-natural substitutes, chemical changes on the main chain or side chains, cyclization, glycosylation, phosphorylation, and PEG coupling [4,9]. Additionally, in order to surpass limitations in APCs’ use, scientists are investigating the use of alternative delivery systems like, encapsulation by liposomes, quantum dots and association with nanoparticles [13]. Indirect uses of APCs include targeted delivery, cancer vaccines, and diagnostic tools [14,15,16].

Among these approaches, nanoparticle-based delivery systems are particularly attractive because they can simultaneously protect peptides from degradation and improve their tumor-selective accumulation [17]. Particles in the range from 1 to 100 nm that consist of more than 50% of a synthetic or natural material are called nanoparticles and are studied for their use as delivery systems [14,15,16]. Iron oxide nanoparticles (IONPs) are inorganic nanoparticles composed of ferromagnetic materials that exhibit unique magnetic properties [16,18].

IONPs form aggregates in a magnetic field and form a stable suspension when the magnetic field is removed. IONPs have a low production cost, are environmentally safe, and exhibit increased colloidal stability and biological compatibility. IONPs, particularly SPIONs, due to their characteristics, have been used in a variety of biological applications, including diagnostics, imaging, hyperthermia, magnetic-field-based separation, modulation of cell growth, biosensing, tissue healing, and drug delivery [16,19,20,21]. Notably, the ability of IONPs to act simultaneously as therapeutic carriers and imaging agents provides a foundation for theranostic strategies in oncology.

In cancer treatment, IONPs enhance drug activity in combination with therapy (i.e., chemotherapeutic drugs) or as hyperthermia agents [16,20,22,23]. Furthermore, drug resistance in cancer could be overcome if IONPs are used for delivery, due to decreased efflux-pump activity at the cell surface, leading to a higher drug concentration in tumor cells [24]. Various pharmacological components may be effectively attached to IONPs. Techniques of interaction include adsorption diffusion in the polymer matrix, encapsulation in the nucleus, electrostatic attraction, and covalent attachment to the outer layer [16]. IONPs coatings include organic and inorganic compounds. Organic compounds include small molecules, surfactants, and biomolecules such as enzymes, antibodies, proteins, biotin, human/bovine albumin, avidin, hydroxyethyl starch, and polypeptides (R), which are highly biocompatible. Inorganic coatings (silica, carbon, metals, metal oxides and sulfides) can connect biological compounds at the surface of IONPs, leading to an increase in their antioxidant properties, stability and in some cases (metal oxides) increase the generation of heat by IONPs in hyperthermia treatment [21,25,26].

These properties make IONPs particularly suitable platforms for peptide functionalization, where peptides confer biological specificity and IONPs provide delivery, imaging, and magnetic responsiveness.

## 2. Iron Oxide Nanoparticles in Oncology

Iron oxide nanoparticles, in particular superparamagnetic iron nanoparticles subtype that exhibit superparamagnetism at core sizes typically below ~20 nm (SPIONs), have found wide application in oncology as diagnostic and therapeutic agents [27]. The advantage of superparamagnetic particles is the disappearance of magnetization when the effect of an external magnetic field is removed, thereby avoiding aggregation in biological environments [28]. Iron oxide nanoparticles are composed of magnetite (Fe_3_O_4_), maghemite (γ-Fe_2_O_3_), or hematite (α-Fe_2_O_3_) and, in addition to the standard spherical shape, they can be obtained in the form of cubes, rods, disks, flowers, hollow spheres, worms, stars, or tetrapods [29]. An essential characteristic of such particles is their size. For example, with a core size of 10–20 nm, particles exhibit superparamagnetic properties (SPIONs) [30]. Despite their small size, nanoparticles exhibit high magnetic susceptibility and, when a magnetic field is applied, provide a stronger and faster magnetic response. Particle size also influences their biodistribution and circulation time in the bloodstream. Particles < 10 nm can quickly penetrate the tumor and are also quickly eliminated from it. Particles 10–100 nm in size are optimal for tumor accumulation and circulation time in the bloodstream. Macrophages actively capture particles >100 nm in the liver and spleen and have a short circulation time [31].

To increase colloidal stability, prolong circulation, reduce toxicity, and avoid sequestration by the mononuclear phagocytic system, IONPs are coated with various materials. These coatings can be broadly categorized as follows: (i) Polymeric shells (e.g., PEG, PVP, PVA, chitosan, dextran, hydroxyethyl starch) enhance hydrophilicity and stealth properties [32,33]. (ii) Lipid-based and surfactant coatings (e.g., oleic acid, polyarabic acid) may improve biocompatibility but can reduce aqueous solubility if overly hydrophobic [34,35]. (iii) Protein coatings (e.g., bovine serum albumin, human serum albumin) can modulate receptor interactions, cellular uptake, and pharmacokinetics [36]. Notably, upon intravenous administration, nanoparticles rapidly adsorb plasma proteins, forming a ‘protein corona’ that critically determines their biological identity, targeting capability, and clearance pathways [37,38]. (iv) Inorganic coatings (silica, carbon, gold, CaCO_3_) improve chemical stability, enable further functionalization, or provide additional imaging/therapeutic functions [39,40,41,42]. (v) Biomimetic coatings (cell membranes from macrophages, stem cells, or platelets) exploit natural tropism for tumor targeting and immune evasion [43,44,45].

Core–shell architectures, in which the iron oxide core is coated with another material (e.g., gold, platinum, silica, or CaCO_3_), can impart additional functionalities such as plasmonic properties, catalytic activity, or pH-responsive drug release [41,42].

It is important to distinguish between the general magnetic properties of IONPs (e.g., superparamagnetism, which enables magnetic targeting and hyperthermia) and their role as MRI contrast agents. The latter depends on their paramagnetic behavior, which influences the relaxation times (T_1_ or T_2_) of surrounding water protons. Most coated IONPs act as T_2_ (negative) contrast agents due to their strong magnetic susceptibility; however, ultrasmall (<5 nm) or specially engineered IONPs (e.g., γ-Fe_2_O_3_ with low crystallinity) can serve as T_1_ (positive) contrast agents [46,47,48].

**Figure 1:**
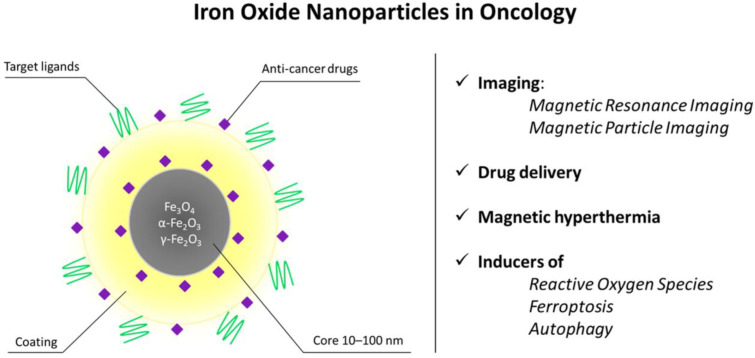
Structural features and biomedical applications of iron oxide nanoparticles (IONPs) in oncology. Schematic representation of an IONP composed of a magnetic Fe_3_O_4_/Fe_2_O_3_ core (10–100 nm) surrounded by a stabilizing coating and functional moieties, including targeting ligands and anti-cancer drugs. The magnetic properties of the core enable non-invasive imaging by magnetic resonance imaging (MRI) and magnetic particle imaging (MPI), while the surface can be engineered for controlled drug delivery and magnetic hyperthermia. Upon cellular uptake, IONPs may also generate reactive oxygen species and promote regulated cell death pathways such as ferroptosis and autophagy. Together, these features support the use of IONPs as multifunctional theranostic platforms in cancer treatment.

The magnetic properties of iron nanoparticles enable them to serve as T2 and T1 contrast agents for magnetic resonance imaging (MRI), a noninvasive, radiation-free method for visualizing soft tissues in vivo [49]. This method utilizes the magnetic properties of hydrogen atoms found in human tissues, coupled with a powerful magnetic field. Superparamagnetic particles act as T2 contrast agents (negative contrast, tissues with accumulated SPIONs appear darker on MRI images) [50], while ultrasmall SPIONs with a nucleus size <5 nm act as T1 (positive contrast, bright signal) [46,47]. For T1 contrast, maghemite (γ-Fe_2_O_3_) particles are preferred due to their lower crystallinity, smaller nucleus size, and, therefore, better positive contrast [48]. Figure 1 shows the schematic depiction of the biomedical applications of IONPs in oncology.

The targeted delivery of IONPs to the tumor site can be achieved by applying an external magnetic field or by modifying the particles’ surfaces with targeting ligands (antibodies, aptamers, folic acid, peptides) [51].

IONPs can also simultaneously act as carriers of antitumor substances and imaging agents, which allows real-time tracking of particle distribution, assessment of tumor accumulation, and monitoring of therapy effectiveness of resulted theranostic remedies [52,53]. The ability of magnetic particles to heat up under the influence of an external alternating magnetic field forms the basis of preparations for magnetic hyperthermia [52,53,54,55]. Magnetic hyperthermia can also serve as an adjuvant to chemotherapy and radiation therapy [56].

Another feature of iron particles is their capability to independently reduce the viability of tumor cells due to the formation of reactive oxygen species [30,31,57]. These radicals cause nonspecific damage to lipids of cell membranes, proteins, and DNA, leading to oxidative stress and ultimately to cell death. Moreover, treatment of Caco-2 colon cancer cells with a combination of IONPs and 5-FU at 5-FU concentrations below the threshold decreased cell viability and total antioxidant capacity (TAC), whereas total oxidant status (TOS) was increased compared with cells treated with 5-FU alone. This study shows that IONPs can exert a synergistic cytotoxic effect with chemotherapeutics at concentrations below the active drug threshold levels [58].

The use of ferroptosis, a non-apoptotic, iron-dependent form of cell death characterized by lipid peroxidation, is another promising approach for cancer therapy [59]. At the same time, IONPsare characterized by their ability to induce the process of autophagy [60]. Indeed, in cancer therapy, iron nanoparticles can improve tumor cell sensitivity to hyperthermia or ferroptosis induction via autophagy [61,62].

Many drugs based on iron nanoparticles are approved by the FDA and used as contrast agents: Ferumoxytol (Feraheme^®^ (USA), Rienso^®^ (EU)), Ferumoxsil (Lumirem^®^ (USA), GastroMARK^®^ (EU)), Ferumoxide (Feridex I.V. (USA), Endorem™ (EU)) and others [62].

Despite their therapeutic potential, bare IONPs can induce significant toxicity, primarily through the generation of reactive oxygen species (ROS) via Fenton-like reactions [18,19,63]. Uncontrolled ROS production can cause oxidative damage to lipids, proteins, and DNA, leading to inflammation, organ toxicity (especially in the liver and spleen), and potential long-term accumulation [19,22,50,63]. This intrinsic toxicity has limited the clinical use of uncoated IONPs. Consequently, surface functionalization is not merely an option but a necessity for biomedical applications. Appropriate coatings (e.g., PEG, polysaccharides, proteins) can shield the reactive iron surface, reduce nonspecific protein adsorption, improve biocompatibility, and direct nanoparticles to target tissues while minimizing off-site damage [32,36]. Therefore, the design of peptide-functionalized IONPs must always consider coating strategies that balance targeting efficacy with safety [32,33,36]. To contextualize the clinical position of IONPs within the broader landscape of nanomedicine, Table 1 provides a comparative overview of IONPs against other clinically approved nanocarriers, highlighting advantages in targeting, imaging, and biodegradability, as well as challenges such as potential iron accumulation.

## 3. Therapeutic Peptides Used in IONP Functionalization

Therapeutic peptides constitute one of the most versatile classes of ligands for nanoparticle engineering due to their intrinsic selectivity, structural diversity, and compatibility with chemical conjugation strategies. When coupled with IONPs, these peptides can guide nanocarriers to distinct tumor microenvironments, facilitate internalization through energy-dependent or -independent pathways, or enhance therapeutic efficacy by engaging specific cell-surface receptors. Based on their biological roles and modes of interaction, the most widely used peptides for IONP functionalization can be broadly divided into tumor-homing peptides, extracellular matrix (ECM)–binding peptides, cell-penetrating peptides, and receptor-targeting peptides.

Peptide selection can be adapted to tumor-specific biomarkers, enabling precision targeting [17,23,63]. For instance, αvβ3 integrin overexpression in glioblastoma and breast cancer favors RGD-based IONPs [49,63]. CD13 (aminopeptidase N) is highly expressed in ovarian tumor vasculature, making NGR peptides ideal for these malignancies [43,63]. HER2-positive breast cancers are effectively targeted by HER2-binding peptides (e.g., AHNP) [13,17], while transferrin receptor (TfR) overexpression in brain tumors supports the use of T7 peptide for blood–brain barrier penetration [17,23]. Such biomarker-driven differentiation, potentially guided by pretreatment biopsy or imaging, can maximize therapeutic efficacy and minimize off-target effects [17,23,63,64,65,66,67,68,69,70,71,72,73,74,75,76]. To directly compare the performance of peptide-functionalized iron oxide nanoparticles with their non-functionalized counterparts, representative preclinical studies are summarized in Table 2.

### 3.1. Tumor-Homing Peptides

Tumor-homing peptides are among the earliest and most extensively studied categories of targeting ligands used in nanomedicine. The prototypical peptide of this class is the Arg-Gly-Asp (RGD) motif, identified for its high affinity toward αvβ3 and αvβ5 integrins, which are abundantly expressed in angiogenic vasculature and in many aggressive tumor phenotypes [66,77,78,79,80]. Conjugation of RGD to IONPs has therefore been widely explored to enhance tumor retention and improve intracellular accumulation. Examples include c(RGDyK)-modified IONPs designed for selective integrin αvβ3 targeting [74], PEG-b-AGE-coated RGD-IONPs for glioblastoma and breast cancer models [75], and RGD-IONPs employed as MRI contrast agents to monitor biochemical responses to therapy [81]. Multifunctional constructs such as RGD-IONP/CAPE further illustrate the capacity of integrin-directed nanoparticles to deliver therapeutic payloads while maintaining tumor specificity [82].

A parallel tumor-homing sequence, Asn-Gly-Arg (NGR), binds a tumor-associated isoform of aminopeptidase N (APN/CD13), enabling selective targeting of angiogenic vessels [67,68,74,75,78,79,80,81,82,83,84,85]. NGR-based nanocarriers have been developed for both fluorescence and MR imaging, including cNGR-SPIOs for visualization of angiogenesis [84], Cy5.5-NGR-Fe_3_O_4_ nanoparticles for ovarian cancer imaging [43], and dual-functional RGD_10_–NGR_9_ USPIO constructs [85].

A more advanced peptide in this category is iRGD (CRGDK/RGPD/EC), which first binds integrins and then, following proteolytic cleavage, exposes a C-end Rule (CendR) motif that interacts with neuropilin-1 to trigger active tissue penetration [68,86]. This mechanism enables iRGD to enhance tumor permeability to both free drugs and nanoparticle-bound therapies. Consequently, several iRGD-modified IONP platforms have been reported, including co-administered SPION formulations [87], iRGD-nanoworm systems [88], iRGD-decorated IO@PNP carriers [89], bcc-USINPs functionalized with iRGD for ferroptotic activity [71], gadolinium/iron hybrid iRGD–nanoparticles [90], and hierarchical AuNP//Co-MION@CMC_iRGD nanoassemblies for multimodal therapy [91].

### 3.2. ECM-Binding Peptides

The tumor ECM is frequently dense, crosslinked, and compositionally altered, posing a substantial barrier to uniform drug penetration [92,93,94]. The interactions between the ECM and nanoparticles are governed by the structural features of the intercellular matrix as well as the physicochemical properties of the nanoparticles and can result in either detrimental or beneficial effects [72]. ECM-binding peptides have therefore been employed to direct nanoparticles into stromal compartments or to modulate the tumor matrix itself. The CSG peptide, which targets laminin and nidogen-1, has been used to guide IONPs into stromally enriched tumor regions [73]. Additional ECM-interacting designs include c(RGDyK)-modified HAase-IONPs loaded with doxorubicin, where hyaluronidase facilitates localized stromal degradation to enhance intratumoral drug distribution [95]. Non-enzymatic ECM-targeted formulations include HA-coated IONPs that exploit CD44 and LYVE-1 expression for selective uptake in HA-rich malignancies [96], PLL-coated magnetic nanoparticles interacting with heparan sulfate proteoglycans [76], and USPIO-collagelin constructs enabling collagen-specific imaging and delivery [97]. Collectively, ECM-binding peptides expand the spatial reach of IONPs in tumors where fibrosis or matrix rigidity limit therapeutic efficacy.

### 3.3. Cell-Penetrating Peptides

Cell-penetrating peptides (CPPs) represent a unique class of ligands capable of translocating across cellular membranes and enabling efficient intracellular delivery of nanoparticle systems [98]. The canonical CPP TAT (YGRKKRRQRRR) is highly cationic and interacts electrostatically with negatively charged membranes, promoting both endocytic uptake and endosomal escape. TAT-functionalized dextran-coated IONPs have been shown to enhance intracellular accumulation [70], while TAT-modified cisplatin-loaded SPIONs increased therapeutic efficacy against cisplatin-resistant nasopharyngeal carcinoma cells [99].

Another relevant CPP is Pep42 (CTVALPGGYVRVC), which targets GRP78, a stress-induced chaperone overexpressed on the surface of various tumors. Pep42-IONP systems, such as Fe_3_O_4_-βCD-Pep42-DOX, demonstrated efficient receptor-mediated internalization and enhanced cytotoxicity [100]. Arginine-rich CPPs, including R8, R10, and R11, are capable of direct membrane penetration and are frequently exploited for nanoparticle delivery: R10-modified CTX-IONPs exhibit siRNA delivery capabilities in glioblastoma [101], while R11-functionalized SPIONs have been used for bladder cancer targeting [102]. Additional specialized CPPs, such as LN1 (CTGTPARQC) for prostate cancer [69] and NFL, which targets glioma cells via magnetic pSiNRs [103], further expand the application landscape of CPP-IONP constructs.

### 3.4. Receptor-Targeting Peptides

Receptor-targeting peptides exploit the overexpression of specific receptors on cancer cells to enhance the precision of nanoparticle-based diagnostics and therapies. Several peptide ligands have been used for this purpose. Among the most widely studied are EGFR-targeting peptides, including GE11, used to functionalize SPIONs for enhanced EGFR-mediated uptake [104], and P22, which has been incorporated into dendronized nanocarriers for targeted delivery [105]. Similarly, peptide ligands recognizing HER2/neu have been coupled to IONPs to facilitate targeted paclitaxel delivery to HER2-overexpressing cancers [106].

Beyond the ERBB family, tumor-associated antigens have also been targeted using optimized peptide–nanoparticle architectures designed to improve specificity via linker or spacer engineering [107]. Peptides targeting IGF-IR have been conjugated to IONPs for pancreatic cancer theranostics [108], while transferrin receptor (TfR)–binding peptides such as T7 have been employed for transport across the blood–brain barrier and delivery into medulloblastoma tissues [75,109]. Receptor-targeting peptides also include relaxin (RLX), which binds RXFP1 and has been used to create RLX-SPION systems enabling stromal modulation and enhanced gemcitabine penetration in pancreatic tumors [110]. Finally, FGF2-functionalized SPIONs demonstrate the ability to target FGFR-expressing cells and exhibit antifibrotic activity [111]. A summary of therapeutic peptides used in IONP functionalization is presented in Table 3.

## 4. Applications of Peptide-Functionalized Iron Oxide Nanoparticles in Oncology

Peptide-functionalized iron oxide nanoparticles (IONPs) represent a versatile platform for cancer theranostics, providing highly specific tumor targeting while enabling multimodal imaging and controlled delivery of therapeutic payloads. Their clinical promise lies in the dual ability to achieve selective accumulation within tumors, through peptide–receptor recognition or peptide-mediated tissue penetration, and to act as carriers for chemotherapeutics, nucleic acids, contrast agents, and hyperthermic energy. The magnetic properties of IONPs further expand their utility, enabling MRI and MPI, externally guided magnetic targeting, and magnetically induced thermal ablation. When combined with targeting peptides such as RGD, NGR, iRGD, GE11, T7, or CPPs, IONPs display enhanced selectivity, deeper tumor penetration, and improved pharmacokinetics compared with nonfunctionalized nanoparticles [69,74,81,82,84,85,87,88,96,108,109]. Below, we summarize the therapeutic and diagnostic applications of peptide–IONP systems, integrating drug delivery, imaging, tumor penetration, ECM modulation, and multimodal therapeutic strategies [69,85,109].

### 4.1. Targeted Delivery of Chemotherapeutic Agents

A major application of peptide–IONPs is the selective delivery of chemotherapeutic drugs to malignant tissues. By exploiting the overexpression of integrins, growth factor receptors, or tumor-associated antigens, peptide-decorated IONPs enhance cellular uptake and intratumoral retention. Among the earliest examples are RGD-modified IONPs, which recognize αvβ3/αvβ5 integrins overexpressed in angiogenic vasculature. c(RGDyK)-coated IONPs accumulated in integrin-rich tumors and improved drug delivery [74]. PEG-b-AGE–coated, RGD-functionalized IONPs enhanced selective uptake in glioblastoma and breast cancer [75], while RGD-IONP/CAPE particles enabled αvβ3-mediated internalization and enhanced apoptosis in myeloma models [82]. RGD-based targeting has also been used in multimodal carriers combining chemotherapy and imaging [81].

NGR-functionalized IONPs have shown efficient uptake in CD13-positive angiogenic vessels [84,85]. Their tumor vasculature specificity has been exploited to deliver doxorubicin and other small molecules with reduced off-target accumulation.

iRGD-functionalized IONPs represent a significant advancement in targeted delivery due to their dual integrin binding and neuropilin-1–mediated tissue penetration [68,86]. iRGD-SPIONs have been shown to penetrate deeply into tumor parenchyma, as demonstrated in SPION co-administration models [87], iRGD-conjugated nanoworms [88], iRGD–IO@PNP systems [89], and bcc-USINPs enabling DOX delivery [71]. Multimodal systems such as ipGdIO-Dox allow simultaneous contrast enhancement and targeted chemotherapy [90]. Hybrid constructs such as AuNP//Co-MION@CMC_iRGD achieve deep tumor penetration, integrated imaging, and synergistic therapy [91].

Beyond tumor-homing peptides, receptor-specific ligands have been used to target chemotherapeutic loading to tumor cells. IGF-1–modified IONPs increased accumulation of DOX in IGF-IR–positive pancreatic cancers [108], and HER2-targeting peptides enabled HER2-specific IONP–paclitaxel delivery [106]. In brain tumors, T7 peptide-functionalized Fe_3_O_4_@T7/AS1411/DTX&SKN-M enhanced drug penetration across the blood–brain barrier and demonstrated improved activity against medulloblastoma [109]. Drug loading strategies include covalent conjugation, electrostatic interaction, or encapsulation within surfactant or polymeric shells [113,114,115,116,117,118,119]. DOX has been loaded into particle shells or conjugated to surfaces through cleavable linkers [118,119]. Dual-drug formulations, including paclitaxel–curcumin SPIONs [120] and curcumin–doxorubicin hybrids [121], demonstrated enhanced therapeutic synergy. Altogether, peptide-functionalized IONPs improve the selectivity and efficacy of chemotherapeutic delivery by leveraging receptor-mediated uptake and enhanced tumor penetration.

The targeting advantage provided by peptide functionalization is quantitatively demonstrated in direct comparative preclinical studies. For instance, in glioblastoma models, RGD-conjugated IONPs showed a 3- to 5-fold higher tumor accumulation than non-targeted IONPs, as quantified by MRI and ex vivo iron assays [81]. Similarly, iRGD-decorated IONPs penetrated 50% deeper into pancreatic tumor spheroids compared to their non-targeted counterparts [89]. In vivo, T7-peptide-modified IONPs crossed the blood–brain barrier and accumulated in medulloblastoma at levels 4-fold higher than unmodified particles [109]. These examples underscore that peptide conjugation substantially enhances tumor-specific delivery and penetration, overcoming the limitations of passive EPR-based accumulation.

### 4.2. Delivery of Nucleic Acids

Peptide–IONP hybrids have also been applied to the delivery of nucleic acids, including siRNA, antisense oligonucleotides, plasmids, and microRNA mimics. Delivery efficiency is enhanced by the presence of cell-penetrating peptides (CPPs) or receptor-binding peptides that facilitate intracellular trafficking. CTX/R10–SPION constructs achieved efficient delivery of siRNA into glioblastoma cells via combined chlorotoxin targeting and arginine-rich CPP-mediated internalization [101]. TAT-functionalized IONPs have been used to transport DNA and RNA molecules across cell membranes [70]. R11-modified SPIONs exhibited improved transfection efficiency in bladder cancer due to arginine-mediated internalization [102]. These examples highlight the adaptability of peptide–IONPs as carriers for genetic therapies in oncology.

### 4.3. Imaging Applications (MRI, MPI, Optical Imaging)

Thanks to their superparamagnetic properties, IONPs are well-established MRI contrast agents, and peptide functionalization improves tumor localization and imaging sensitivity. RGD-modified SPIONs have been employed as T_2_ MRI contrast agents, enabling visualization of αvβ3-positive glioblastoma and monitoring of therapy response [47,74,75,81]. NGR-SPIOs enhanced detection of angiogenic vasculature [84], and Cy5.5–NGR–Fe_3_O_4_ nanoparticles enabled dual MRI/fluorescence imaging of CD13-positive ovarian tumors [43]. Dual RGD–NGR USPIOs combined both ligands to maximize tumor accumulation and improve MRI contrast performance [85].

Ultrasmall SPIONs (<5 nm), particularly those composed of maghemite (γ-Fe_2_O_3_), act as T_1_ contrast agents and provide bright-signal MRI. Their small core reduces crystallinity and enhances T_1_ relaxivity [46,47,48]. Ultra-small silicon-coated SPIONs showed excellent biocompatibility in vitro and in vivo and were successfully applied for imaging of the heart, liver, kidneys, and bladder [47]. Iron-based contrast agents are emerging as attractive alternatives to gadolinium chelates due to lower toxicity and longer circulation times [64].

Peptide-functionalized IONPs have also been explored as tracers for magnetic particle imaging (MPI), a modality that detects magnetic nanoparticles directly with no background signal [65,122]. RGD, NGR, and iRGD enhance MPI sensitivity by promoting tumor-specific nanoparticle accumulation. Optical imaging capabilities can be integrated by attaching fluorescent dyes to peptide–IONP systems, as demonstrated by Cy5.5-labeled NGR–Fe_3_O_4_ nanoparticles [112]. Peptide targeting improves fluorescence signal localization in vivo by reducing off-target distribution. Altogether, peptide–IONP platforms provide multimodal imaging capabilities that enhance tumor delineation and support image-guided therapy.

### 4.4. Tumor Penetration and Overcoming Biological Barriers

Poor vascularization and a dense ECM [123] restrict the diffusion of most nanomedicines into solid tumors [32,94]. Peptide-functionalized IONPs—especially those modified with iRGD—address this limitation by exploiting neuropilin-1–mediated trans-tissue transport [68,86]. iRGD-modified SPIONs demonstrated deep tumor penetration across multiple platforms, including nanoworm-based IONPs [88], IO@PNP nanoparticles [89], bcc-USINPs [71], and hierarchical metal–iron oxide hybrids [91]. These systems enhanced the uniformity of drug distribution and improved therapeutic outcomes, particularly in poorly perfused tumors.

ECM-binding peptides also contribute to enhanced penetration. CSG-functionalized nanoparticles localized preferentially within tumor stroma [73]. Hyaluronidase-linked RGD–IONP/DOX degraded hyaluronic acid and increased intratumoral spread [95]. HA-coated IONPs utilized CD44 and LYVE-1 overexpression to traverse HA-rich tumors [96]. Collagen-targeting collagelin-USPIOs further expanded ECM-directed imaging and delivery options [97]. These findings underscore the utility of peptide-functionalized IONPs in addressing physical barriers that limit drug penetration in solid tumors.

### 4.5. ECM Modulation and Stromal Remodeling

Peptide-functionalized IONPs can directly modulate the tumor microenvironment to improve therapeutic outcomes. Tumors with extensive fibrosis or stromal desmoplasia, such as pancreatic cancers, are particularly amenable to such strategies. Relaxin-functionalized SPIONs reduced collagen deposition and stromal stiffness by targeting RXFP1 receptors on fibroblasts, facilitating gemcitabine penetration and improving therapeutic response in pancreatic cancer models [110]. Hyaluronidase-linked IONPs reduced intratumoral hyaluronic acid levels, improved perfusion, and enhanced distribution of co-delivered drugs [95]. HA-IONPs enabled selective targeting of HA-rich tumors and contributed to stromal modulation [96]. PLL-modified magnetic nanoparticles interacted with sulfated GAGs to alter stromal distribution [76]. ECM-modulating peptide–IONPs therefore provide a compelling approach to overcoming stromal resistance in desmoplastic tumors.

### 4.6. Magnetic Targeting and Field-Assisted Accumulation

One advantage of IONPs is their responsiveness to externally applied magnetic fields. Peptide targeting can be combined with magnetic guidance to concentrate nanoparticles at the tumor site. Magnetically guided peptide–IONP systems have been shown to increase intratumoral retention, overcome rapid systemic clearance, and enable controlled spatial localization of both imaging and therapeutic payloads. Although peptide targeting provides biological specificity, magnetic targeting enhances physical accumulation, creating a dual-targeting strategy. This combined targeting approach is particularly useful in tumors with low vascular permeability, where passive nanoparticle accumulation is insufficient. 

### 4.7. Multimodal and Synergistic Therapeutic Platforms

A final, rapidly expanding area involves the construction of multifunctional platforms that combine several therapeutic modalities. IONPs are naturally suited for such integration, and peptide functionalization brings an additional level of biological specificity.

Multifunctional peptide–IONP hybrids integrate therapeutic and diagnostic modalities into a single construct. iRGD-modified hybrid particles, such as AuNP//Co-MION@CMC_iRGD, combined deep tissue penetration with chemotherapy and magnetic responsiveness to produce synergistic tumor suppression [91,124]. Various dual-drug SPIONs have demonstrated enhanced cytotoxicity due to their ability to deliver multiple agents simultaneously or sequentially. For example, SPIONs co-loaded with paclitaxel and curcumin [120], or curcumin and doxorubicin [121], achieved superior anticancer effects through complementary mechanisms. Theranostic systems such as ipGdIO-Dox combine imaging and therapy into a single platform [90]. Fluorescence/MRI dual-modality peptide–IONPs further enhance diagnostic accuracy [43]. Complex constructs that unite chemotherapy, magnetic hyperthermia, and ROS-driven ferroptosis have shown powerful synergy. Peptide–IONP hybrids, therefore, represent a flexible and scalable platform that can be tuned to address multiple barriers in cancer therapy, including poor tumor penetration, stromal resistance, limited drug uptake, and cell-intrinsic mechanisms of survival.

**Figure 2:**
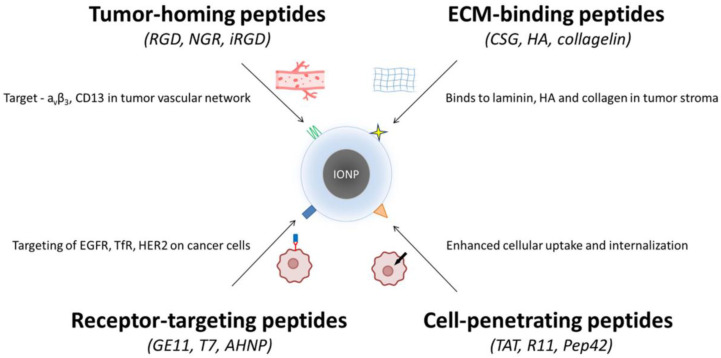
Peptide classes used for functionalizing iron oxide nanoparticles (IONPs). Four major peptide categories are applied to improve the targeting and uptake of IONPs in cancer therapy. (i) Tumor-homing peptides (RGD, NGR, iRGD) bind markers such as αvβ3 integrins and CD13 in tumor vasculature. (ii) ECM-binding peptides (CSG, HA-derived sequences, collagelin) recognize laminin, hyaluronan, and collagen within the tumor stroma. (iii) Receptor-targeting peptides (GE11, T7, AHNP) engage overexpressed receptors on cancer cells, including EGFR, TfR, and HER2. (iv) Cell-penetrating peptides (TAT, R11, Pep42) enhance cellular uptake and intracellular delivery. Together, these peptide groups enable improved selectivity and penetration of IONP-based nanotherapeutics.

These multimodal platforms exemplify the flexibility of peptide–IONP systems and their potential in precision nanomedicine. The classes of peptides used for functionalizing IONPs are depicted in Figure 2.

## 5. Mechanistic Insights into the Biological Activity of Peptide–IONP Hybrids

Peptide-functionalized iron oxide nanoparticles (IONPs) operate through a multilayered series of interactions that begin at the nano–bio interface and progress through membrane engagement, intracellular trafficking, and iron-dependent biochemical reactions. These processes ultimately converge on regulated cell death pathways and tumor microenvironment remodeling. The mechanistic behavior of peptide–IONP hybrids is therefore far more complex than the sum of a targeting ligand and a magnetic nanoparticle; instead, it reflects a carefully orchestrated interplay between peptide architecture, nanoparticle surface chemistry, and cellular physiology. The mechanistic efficacy of peptide–IONP hybrids can be quantified through key parameters governing their interaction with biological systems. These include peptide-receptor binding affinities, cellular uptake efficiency, thresholds for inducing iron-dependent cell death, and imaging performance metrics. Table 4 summarizes representative quantitative data from current studies, providing a reference for the design and evaluation of future constructs.

A central mechanistic determinant is the structural presentation of peptides on the nanoparticle surface. The density, orientation, and conformational freedom of targeting motifs critically shape how they engage with biological structures. Cyclic peptides such as c(RGDyK), for example, maintain their active conformation more reliably on curved IONP surfaces than their linear counterparts, preserving integrin-binding affinity even after conjugation [74]. Likewise, the use of polyethylene glycol (PEG) linkers provides steric spacing from the nanoparticle coating, preventing peptide burial and ensuring solvent accessibility [75]. Multivalent presentation of peptides on IONPs can further amplify binding affinity by creating avidity effects that do not occur with free peptides in solution. Where reported, this avidity can be quantified by apparent dissociation constants in the low-nanomolar range for integrin ligands [e.g., c(RGDyK) Kd ≈ 10 nM measured by microscale thermophoresis] [125], providing a mechanistic basis for stronger cell association than non-targeted IONPs under identical exposure conditions.

When peptide–IONP hybrids encounter cellular membranes, distinct mechanistic pathways begin to unfold depending on the peptide class. Receptor-targeting peptides, such as RGD, NGR, or GE11, bind to integrins, CD13, or EGFR, respectively, initiating receptor clustering and internalization. These interactions can have functional consequences independent of drug payloads. For example, integrin clustering induced by multivalent cRGD–IONPs can reorganize focal adhesion complexes and modulate downstream signaling, subtly influencing cell migration or survival pathways [85]. EGFR-binding peptides such as GE11 promote clathrin-dependent internalization without the mitogenic signaling associated with EGF itself, enabling selective trafficking of nanocarriers into endolysosomal compartments [104]. This “binding-without-activation” concept has been experimentally supported for GE11 (YHWYGYTPQNVI) as an EGFR ligand [126], helping to explain why peptide targeting can increase uptake/trafficking while avoiding growth-factor–like signaling that may confound interpretation in control (non-peptide) systems.

In contrast, cell-penetrating peptides (CPPs) interact with membranes through electrostatic attraction to negatively charged phospholipids and glycosaminoglycans. Poly-arginine motifs facilitate transient membrane destabilization, promoting macropinocytosis or direct cytosolic entry. TAT-modified IONPs exemplify this behavior, as their lysine- and arginine-rich sequences enhance endosomal escape following uptake and promote cytosolic distribution of the nanoparticle and its cargo [70]. An important mechanistic consequence of peptide functionalization is the determination of endocytic sorting routes, which strongly influence intracellular processing of IONPs. Integrin-targeting peptides guide nanoparticles toward clathrin-mediated endocytosis, whereas CPPs favor macropinocytosis or caveolin-associated pathways. Peptides such as Pep42, which bind the chaperone GRP78 frequently overexpressed on cancer cells, direct nanoparticles to specific subdomains of the endoplasmic reticulum and Golgi trafficking networks [100]. These entry routes dictate the rate and location at which IONPs encounter lysosomal acidity and degradative enzymes, ultimately controlling when and where Fe^2+^ ions are released.

Once within endosomes and lysosomes, peptide–IONP hybrids enter the phase of mechanistic activity driven by iron. The acidic lysosomal environment promotes dissolution of the iron oxide core, generating Fe^2+^ ions that participate in Fenton and Fenton-like reactions with hydrogen peroxide. Tumor cells tend to produce elevated basal ROS, making them especially susceptible to the resulting hydroxyl radicals (•OH). When peptide-mediated targeting enhances IONP accumulation in malignant cells, the intracellular iron burden increases substantially, shifting the redox balance toward oxidative damage. This mechanism has been demonstrated with ultrasmall iRGD-modified Fe nanoparticles, which doubled intracellular uptake and triggered lipid peroxidation consistent with ferroptotic cell death [71]. Importantly, this type of comparison directly addresses “with vs. without peptide”: increased uptake is a proximal, quantifiable driver that can explain downstream differences in lipid peroxidation and viability between targeted and non-targeted particles in matched dose/time experiments. Complementary findings in dextran-coated IONPs further highlight how iron accumulation promotes autophagic flux; when this stress exceeds the buffering capacity of the autophagy–lysosome system, autophagic cell death can result [60].

The dominant form of regulated cell death induced by peptide–IONP hybrids depends on nanoparticle size, coating, and the intracellular compartment where Fe^2+^ is released. Rapid dissolution and high Fe^2+^ flux favor ferroptosis, especially when nanoparticles localize in proximity to lipid membranes rich in polyunsaturated fatty acids. Slower iron release or lysosomal destabilization may promote apoptosis through mitochondrial damage. Here, “ferroptosis thresholds” can be operationally defined as the point at which lipid-peroxidation burden surpasses GPX4/antioxidant capacity, typically evidenced by (i) lipid peroxidation probes (e.g., C11-BODIPY), (ii) accumulation of lipid oxidation products (e.g., MDA/4-HNE), and (iii) phenotypic rescue by ferroptosis inhibitors/iron chelation, criteria discussed in recent mechanistic syntheses [59]. The mechanistic balance can be further influenced by peptide choice: CPPs that destabilize lysosomes may accelerate LMP (lysosomal membrane permeabilization), triggering caspase-dependent pathways, whereas receptor-targeting peptides often deliver IONPs into compartments enriched in iron-transport proteins, reinforcing ferroptotic susceptibility.

In addition to biochemical mechanisms, peptide–IONP constructs produce biophysical effects that influence their therapeutic activity. The magnetic core can convert alternating magnetic fields into localized heat, and when peptides target IONPs to ECM components or specific cell populations, this heat is spatially deposited within defined microdomains. Collagen-binding peptide–IONPs are a clear example: by anchoring nanoparticles to collagen fibers, subsequent magnetic hyperthermia can physically soften or partially denature stromal structures, improving drug penetration and altering tumor mechanics [97].

Another layer of mechanism arises from the ability of certain peptides—particularly iRGD—to reconfigure vascular permeability. After integrin binding and proteolytic activation, the C-terminal fragment of iRGD binds neuropilin-1, initiating a transport process that increases tissue penetration for both peptide-bound and co-administered agents. This mechanism explains why simple co-injection of iRGD can enhance tumor uptake of IONPs without direct conjugation [87]. This is a key “transport efficiency” comparator because it demonstrates improved delivery even when the nanoparticle itself is unchanged, e.g., iRGD used as an enhancer rather than a conjugated ligand, consistent with the original iRGD transport mechanism described by Sugahara et al. [127]. The phenomenon represents a rare case in which a peptide can modulate bulk fluid flow across tumor barriers, effectively bypassing the limitations of the enhanced permeability and retention (EPR) effect.

**Figure 3:**
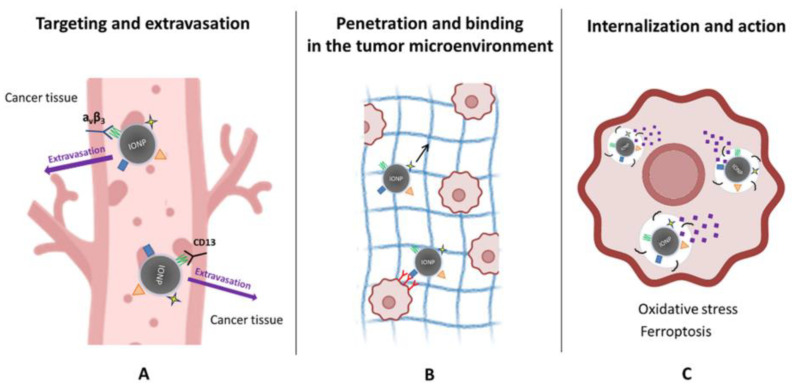
Mechanistic actions of peptide-functionalized iron oxide nanoparticles (IONPs) in tumors. (**A**) Targeting and extravasation: Peptide ligands promote selective binding of IONPs to tumor vasculature by recognizing receptors such as αvβ3 integrins and CD13, facilitating vascular adhesion and extravasation into cancer tissue. (**B**) Penetration and microenvironmental interactions: Following extravasation, peptide–IONPs move through the tumor extracellular matrix (black arrow), where ECM-binding or tumor-penetrating sequences enhance stromal engagement and improve distribution within the tumor microenvironment. (**C**) Internalization and therapeutic action: Cancer cells internalize peptide–IONPs via receptor-mediated or membrane-active pathways. Intracellular processing releases iron ions, driving oxidative stress and lipid peroxidation, ultimately promoting ferroptosis and related cytotoxic mechanisms.

Together, these mechanistic insights reveal that peptide–IONP hybrids behave as dynamic biological systems rather than passive carriers. Their activity reflects a concert of nanoscale structural engineering, receptor biology, iron chemistry, membrane dynamics, and intracellular stress responses. As understanding deepens, these mechanisms can be leveraged to design more refined constructs that exploit ferroptosis thresholds, receptor trafficking peculiarities, or stromal vulnerabilities, thereby enabling therapeutic strategies that are both more selective and more potent than current nanomedicine approaches. The mechanistic aspects of peptide-functionalized iron oxide nanoparticle (IONP) actions in tumors are depicted in Figure 3.

## 6. Translational and Clinical Perspectives

Peptide–IONPs are gradually moving toward translational relevance, driven by the convergence of peptide engineering, nanotechnology, and increasingly, artificial intelligence (AI). What once appeared as highly specialized laboratory constructs now stand out as candidates for precision oncology applications, especially in tumors where delivery barriers or molecular heterogeneity limit conventional therapies. Among these, glioblastoma remains one of the most promising targets. Peptides such as chlorotoxin, T7, and poly-arginine have repeatedly demonstrated the ability to enhance IONP passage in preclinical models [75,101]. Pancreatic cancer, characterized by a dense desmoplastic stroma, has also shown responsiveness to peptide–IONP approaches. Relaxin- and FGF2-functionalized nanoparticles modulate stromal rigidity and enhance gemcitabine efficacy, underscoring the therapeutic value of targeting the tumor microenvironment itself [110]. In ovarian, breast, and colorectal cancers, the reproducible overexpression of receptors such as CD13, integrins, or EGFR supports the use of NGR, RGD, or GE11 peptides for selective accumulation [104,112].

Despite encouraging advances, translation requires addressing both safety and manufacturing challenges. The clinical translation of peptide–IONP hybrids faces several interconnected barriers [17,122]. Regulatory pathways for nanotherapeutics require demonstration of consistent manufacturing (GMP), long-term stability, and detailed pharmacokinetic/toxicokinetic profiles. The FDA and EMA have issued guidelines for nanomedicine approval, emphasizing the need for robust characterization of size, surface charge, peptide density, and in vivo fate [17,122]. Immunogenicity remains a concern, as peptides and nanoparticle coatings may elicit immune responses. Strategies to soften this include PEGylation, use of D-amino acids, or humanized peptide sequences [33,36,66]. Off-target effects can be reduced by employing activating peptides (e.g., protease-cleavable linkers) or dual-targeting systems that enhance tumor selectivity [95,122]. Moreover, the protein corona formed in vivo can obscure targeting peptides; recent advances in stealth coatings (e.g., polysaccharides, biomimetic membranes) help preserve targeting functionality [37,44,96]. Finally, scalable and reproducible manufacturing processes, ensuring uniform peptide conjugation, batch-to-batch consistency, and sterile formulation, are critical for clinical advancement [95,122].

The long-term safety profile of peptide–IONPs depends on their biodegradation and clearance pathways. IONPs are gradually degraded within lysosomes via acidic hydrolysis, releasing Fe^2+^/Fe^3+^ ions that enter the body’s physiological iron pool [18,19,63]. However, repeated administration can lead to progressive hepatic and splenic accumulation, posing a risk for oxidative stress and organ toxicity [19,22,50,63]. Strategies to enhance clearance include the use of ultrasmall particles (<5 nm) for renal excretion [71] or the design of coatings (e.g., dextran, hydroxyethyl starch) that are more readily biodegradable. For patient monitoring, the inherent imaging capabilities of IONPs are a significant advantage [22,32]. MRI or magnetic particle imaging (MPI) can be used non-invasively to track nanoparticle biodistribution and clearance in real-time, enabling personalized dosing [48,65]. In cases of excessive iron burden, adjunctive use of iron chelators (e.g., deferoxamine) could be considered as a safety management strategy [63].

Iron oxide is safer than most inorganic nanomaterials, yet repeated systemic exposure can still result in hepatic or splenic deposition [63]. Peptide functionalization may alter protein corona formation and immunogenicity [128], and the same Fe^2+^-dependent redox reactions that drive ferroptosis in tumors can produce off-target toxicity when biodistribution is suboptimal. Reproducibility of size, surface chemistry, and peptide conjugation remains essential for GMP manufacturing and regulatory approval. One advantage of peptide–IONP platforms is their inherent compatibility with image-guided oncology. Because many formulations provide MRI contrast, clinicians can non-invasively monitor nanoparticle accumulation, allowing for personalized dosing strategies and early identification of non-responders—an approach rarely possible with conventional nanomedicines.

The most rapidly developing dimension of this field, however, is the integration of artificial intelligence. AI is transforming both peptide design and nanoparticle engineering. Recent work has demonstrated that deep learning can autonomously generate or optimize therapeutic peptides, improving structure prediction, stability assessment, and receptor-binding affinity [129,130,131]. Significantly, machine-learning frameworks trained on experimentally validated peptide datasets can predict anticancer activity and selectivity directly from primary sequence features, thereby enabling prioritization of candidate peptides before synthesis and nanoparticle conjugation [132]. These tools allow researchers to explore vast sequence spaces that would be impossible to navigate experimentally, potentially yielding novel tumor-targeting or tumor-penetrating peptides suitable for IONP functionalization.

Beyond activity prediction, AI-guided peptide design increasingly incorporates developability-related parameters, including proteolytic stability, amphipathicity, and binding robustness, which are critical for maintaining peptide function following immobilization on nanomaterial surfaces. Recent comprehensive analyses of machine-learning strategies for peptide drug discovery highlight how AI can integrate sequence, structural, and physicochemical constraints to propose peptides optimized for therapeutic applications [133,134]. AI is also reshaping nanomedicine more broadly. Recent large-scale machine learning analyses have uncovered design patterns underlying successful inorganic nanoparticle formulations in cancer therapy [133]. Machine learning models have begun to predict biodistribution and tumor delivery in vivo, an essential step toward rational nanoparticle design [134]. Emerging frameworks now integrate nanoparticle geometry, surface chemistry, and biological response to guide more efficient discovery pipelines [135]. Even at the mechanistic level, AI is being used to model how nanoparticles enter solid tumors and navigate stromal barriers [136].

Taken together, these advances support a practical AI-assisted design logic for peptide–IONPs, in which AI-guided peptide selection and optimization [129,130,131,132] can be combined with data-driven prediction of nanoparticle delivery, tumor accumulation, and penetration [133,134,135,136].These developments suggest that peptide–IONP systems may increasingly be engineered computationally prior to synthesis, thereby narrowing the gap between design and clinical validation. This perspective aligns with broader trends in AI-driven drug discovery, where computational pipelines are reshaping candidate selection, optimization, and early-stage preclinical assessment [137].

Looking forward, the synergy between peptide engineering, targeted nanotherapy, and AI-enabled design offers a compelling roadmap for translation [129,130,131,133,134,135,136,137]. Recent advances in AI-driven peptide discovery already demonstrate how deep learning can autonomously optimize peptide sequences for stability, receptor affinity, and structural robustness [129,130,131]. The near-term clinical opportunities for peptide–IONP systems will likely arise in biomarker-selected populations, such as αvβ3-positive glioblastoma or CD13-positive ovarian cancer, where targeted nanoparticles can be combined with MRI-based tracking to verify intratumoral accumulation in real time [48,49,112]. Parallel advances in nanomedicine informatics are equally important: large-scale machine learning analyses now reveal design principles governing successful inorganic nanoparticle formulations [133] while predictive models can estimate biodistribution and tumor delivery directly from physicochemical features [134]. Complementary computational frameworks show how nanoparticle geometry and surface chemistry influence tumor penetration and therapeutic efficiency [135,136]. As these computational tools continue to mature, peptide–IONP nanomedicine may evolve into a truly adaptive platform in which targeting sequences, nanoparticle coatings, and dosing regimens are intelligently optimized for each tumor’s molecular and stromal landscape [133,134,135]. This alignment of high-precision biology with AI-driven intelligent design reflects a larger trend across drug development [137], and may ultimately define the next generation of oncologic nanotherapies.

## 7. Conclusions and Future Directions

The strategy of combined application of iron oxide nanoparticles with immobilized therapeutic peptides represents a new step in evolution of the approaches to cancer therapy. This review summarized and justified the benefits for this synergy: peptides provide targeting specificity and tumor-penetrating capabilities, while IONPs provide a versatile platform for drug delivery, hyperthermia, and diagnostic imaging. More than the sum of their components’ separate effects, these combined systems can effectively modulate the tumor microenvironment and engage novel cell death pathways like autophagy and ferroptosis, opening up new therapeutic strategies for resistant cancers.

Despite these advances, several critical challenges remain that must be addressed to enable successful clinical translation of peptide–IONP systems. The future development of this field lies in deepening our understanding and expansion of the functional features of these systems. First, there is an obvious need for more mechanistic studies that will move beyond simple efficacy demonstrations to reveal exact intracellular signaling cascades and trafficking pathways activated by peptide–IONP binding and internalization. Understanding how these combined conjugates influence iron metabolism, lysosomal function, and immunogenic cell death will be crucial for optimizing their design, properties and efficiency.

The second key point is the study of multifunctional peptides. Future studies should utilize peptides that combine cell penetration, targeting and even therapeutic (for example, pro-apoptotic) sequences into a single molecule, conjugated to IONPs which themselves induce ferroptosis. This approach will give us a new generation of high-effective theranostic systems. In parallel, translational progress will depend on addressing regulatory, manufacturing, and standardization challenges.

Third, the clinical translation of these nano-platforms must be accelerated through robust translational trials. Initial clinical studies should focus on well-defined patient populations with validated biomarker expression, using peptide–IONPs as neoadjuvant therapies or in recurrent localized diseases, when their theranostic capabilities can be fully utilized.

The next 3–5 years will be pivotal for advancing peptide–IONP hybrids toward clinical translation. Key priorities include the following: (i) Short-Term (1–3 years): Optimize peptide-linker chemistry for stable conjugation; establish GMP-compliant manufacturing protocols; and conduct comprehensive toxicology studies in large animals. (ii) Mid-Term (3–5 years): Initiate Phase I/II trials in biomarker-selected patients (e.g., αvβ3+ glioblastoma, CD13+ ovarian cancer); integrate AI for patient-specific peptide design; and develop companion diagnostics for patient stratification. (iii) Long-term (5–9 years): Advance to Phase III trials for specific cancer indications; explore combination with immunotherapy (e.g., checkpoint inhibitors); and implement real-time imaging feedback for adaptive dosing.

Addressing these milestones will require close collaboration among nanotechnologists, clinicians, regulatory experts, and AI specialists.

In conclusion, peptide-functionalized IONPs are now ready to make a significant impact on precision oncology. By successful integration of targeted delivery, multi-modal therapy, and non-invasive imaging, they offer a powerful tool to overcome the longstanding challenges of tumor heterogeneity, drug resistance, and treatment side toxicity. Looking forward, the integration of AI-assisted design strategies and personalized medicine frameworks is expected to further refine peptide selection, nanoparticle optimization, and patient stratification, thereby supporting more rational and individualized therapeutic approaches. In the coming years, we will undoubtedly see a surge in complex developments based on AI and personalized medicine, bringing us closer to the clinical implementation of these “magic bullets” in the fight against cancer.

## Figures and Tables

**Table 1:** Comparative overview of iron oxide nanoparticles (IONPs) and other clinically approved nanocarriers.

Nanocarrier Type	Targeting Capability	Imaging Function	Biodegradability	Clinical Status	Key Limitations
Iron oxide na-noparticles (IONPs)	Yes (peptide/antibody conjugation; integrin- and tumor-penetrating peptides) [51]	Yes (MRI, MPI) [48,64,65]	Slow (via iron metabolism) [18,19,22]	Approved as contrast agents; under investigation for therapy (hyperthermia, drug delivery, theranostics) [48,52,53]	Potential iron accumulation in liver/spleen; RES uptake [19,50]
Liposomes (e.g., Doxil^®^)	Limited (passive accumulation via EPR-like effects) [17]	No (unless labeled) [23]	Biodegradable (phospholipid bilayer) [17]	Approved for chemotherapy (e.g., doxorubicin formulations) [17]	Rapid clearance, limited tumor penetration in solid tumors [17]
Polymeric NPs (e.g., Abraxane^®^)	Yes (surface functionalization, ligand conjugation) [17]	No (unless combined with imaging agents) [23]	Biodegradable (albumin, PLGA-based systems) [17]	Approved for paclitaxel delivery [17]	Batch-to-batch variability; immunogenicity and protein corona effects [17]
Silica/gold NPs	Yes (ligand- or peptide-mediated targeting) [23]	Yes (optical, photoacoustic imaging) [23]	Non-biodegradable [15]	Preclinical/early clinical development [23]	Long-term tissue retention; unclear clearance pathways [15,17]
Dendrimers	Yes (multivalent ligand conjugation) [17]	Possible (via labeling or hybrid systems) [23]	Variable (chemistry-dependent) [17]	Preclinical [17]	Synthetic complexity; toxicity concerns and translational barriers [17]

**Table 2:** Comparative performance of peptide-functionalized versus non-functionalized iron oxide nanoparticles in representative preclinical cancer models.

Peptide (Class)	IONP System	Model (In Vitro/In Vivo)	Key Comparative Findings	Reference
RGD (Tumor-homing)	c(RGDyK)-IONPs vs. plain IONPs	U87MG glioblastoma xenograft (in vivo)	3.5-fold higher tumor accumulation (MRI); 2.8-fold increase in intracellular uptake in vitro	[63,66]
iRGD (Tumor-penetrating)	iRGD-SPIONs vs. non-targeted SPIONs	MDA-MB-231 breast cancer xenograft (in vivo)	50% deeper tumor penetration; 2-fold higher doxorubicin delivery to tumor core	[67,68]
T7 (Receptor-targeting)	T7-IONPs vs. plain IONPs	Medulloblastoma xenograft (in vivo)	4-fold higher brain tumor accumulation; 60% longer survival in treated mice	[69]
GE11 (EGFR-targeting)	GE11-SPIONs vs. non-targeted SPIONs	EGFR-overexpressing A431 xenograft (in vivo)	2.8-fold higher tumor-to-muscle ratio (MRI); 70% greater cellular internalization in vitro	[70]
CSG (ECM-binding)	CSG-IONPs vs. unmodified IONPs	4T1 breast tumor model (in vivo)	3-fold higher stromal accumulation; improved intratumoral distribution measured by MPI	[71]
TAT (Cell-penetrating)	TAT-dextran-IONPs vs. dextran-IONPs	A549 lung cancer cells (in vitro)	4-fold increase in cellular uptake; 3-fold higher cytotoxicity when loaded with cisplatin	[72,73]
NGR (Tumor-homing)	NGR-USPIO vs. plain USPIO	HT-1080 fibrosarcoma xenograft (in vivo)	2.5-fold higher angiogenic vessel targeting; enhanced MRI contrast in tumor periphery	[74,75]
R11 (CPP)	R11-SPIONs vs. unmodified SPIONs	Bladder cancer (MB49) model (in vivo)	3.2-fold higher tumor-specific uptake; 40% greater transfection efficiency with siRNA delivery	[76]

**Table 3:** Classes of therapeutic peptides used in IONP functionalization.

Peptide Class	Representative Peptide(s)	Formulation	Reference
Tumor-homing peptides	RGD	IONPs coupled with c(RGDyK) RGD-conjugated PEG-b-AGE coated IONP RGD-IONP as contrast agents in MRI RGD-IONP/CAPE	[74] [81] [82]
	NGR	SPIOs labeled with cyclic NGR peptide (cNGR) Cy5.5-NGR-Fe3O4 NP RGD10-NGR9 targeted USPIO nanoparticles	[84] [112] [109]
	iRGD	iRGD co-administration with SPION iRGD-NWs iRGD decorated IO@PNP iRGD-bccUSINP ipGdIO-Dox AuNP//Co-MION@CMC_iRGD	[87] [88] [89] [71] [90] [91]
ECM-binding peptides	CSG	CSG-IO-NP	[73]
	Hyaluronidase	c(RGDyK)-HAase-IONP/DOX	[95]
	Hyaluronic acid	HA-IONP	[96]
	Poly-l-lysine	PLL-MNP	[76]
	Collagelin	USPIO-POPEG-Collagelin NP	[97]
Cell-penetrating peptides	TAT	TAT-functionalized dextran-coated IONPs TAT-modified cisplatin-loaded SPIONs	[70] [99]
	Pep42	Fe_3_O_4_-ßCD-Pep42-DOX	[100]
	Polyarginine	NP-CTX-R10-siRNA R11-functionalized SPION	[101] [102]
	LN1	LN1-functionalized TMNP	[69]
	NFL	NFL-decorated magnetic-pSiNRs	[103]
Receptor-targeting peptides	GE11 P22	Anti-EGFR GE11 peptide-targeted SPION Anti-EGFR P22 peptide-targeted DNP	[104] [105]
	IGF1	IGF1R-targeted IONP	[108]
	T7	Tf-conjugated PEG-b-AGE coated IONP Fe_3_O_4_@T7/AS1411/DTX&SKN-M	[109]
	Relaxin (RLX))	RLX-SPION	[110]
	FGF2	FGF2-SPION	[111]

**Table 4:** Quantitative mechanistic parameters of peptide-functionalized IONPs.

Parameter	Typical Range/Value	Example System	Reference
Peptide–receptor affinity (Kd)	10–100 nM (cRGD–αvβ3)	c(RGDyK)-IONPs	[74]
Cellular uptake enhancement	3–5× (vs. non-targeted)	RGD-IONPs in U87MG glioblastoma	[81]
Tumor penetration depth increase	~50% deeper with iRGD	iRGD-IO@PNP in pancreatic spheroids	[89]
Ferroptosis threshold (lipid peroxidation)	2–3× increase in MDA/4-HNE	iRGD-bccUSINP in 4T1 cells	[71]
Intracellular Fe^2+^ for ferroptosis	50–100 µM	Ultrasmall Fe NPs in tumor cells	[71]
MRI relaxivity (r_2_, mM^−1^s^−1^)	100–300 (SPIONs, 10–20 nm)	Dextran-coated SPIONs	[50]
Hyperthermia temperature (ΔT)	42–45 °C under AMF	Citrate-coated IONPs	[52]

## Data Availability

No new data were created.

## Abbreviations

ACP	Anticancer peptide
AI	Artificial intelligence
APN	Aminopeptidase N
BBB	Blood–brain barrier
CTX	Chlorotoxin
CAPE	Caffeic acid phenethyl ester
CPP	Cell-penetrating peptide
DNP	Dendronized nanoparticle
DOX	Doxorubicin
ECM	Extracellular matrix
EGFR	Epidermal growth factor receptor
EPR	Enhanced permeability and retention
FGFR	Fibroblast growth factor receptor
GAG	Glycosaminoglycan
GMP	Good Manufacturing Practice
GRP78	Glucose-regulated protein 78
HA	Hyaluronic acid
HAase	Hyaluronidase
HCPT	Homocamptothecin
HCC	Hepatocellular carcinoma
HER2	Human epidermal growth factor receptor 2
HSA	Human serum albumin
IGF-IR/IGF1R	Insulin-like growth factor 1 receptor
IONP	Iron oxide nanoparticle
iRGD	Internalizing RGD peptide
LMP	Lysosomal membrane permeabilization
LYVE-1	Lymphatic vessel endothelial hyaluronan receptor 1
MRI	Magnetic resonance imaging
MPI	Magnetic particle imaging
MM	Multiple myeloma
NP	Nanoparticle
PEG	Polyethylene glycol
PLL	Poly-L-lysine
pSiNR	Porous silicon nanorod
RCT	Receptor-mediated clathrin trafficking (optional; remove if unused)
RGD	Arginine–glycine–aspartic acid motif
ROS	Reactive oxygen species
RXFP1	Relaxin family peptide receptor 1
SPION	Superparamagnetic iron oxide nanoparticle
Tf	Transferrin
TfR/TfR1	Transferrin receptor (type 1)
TMNP	Trimagnetic nanoparticle
TME	Tumor microenvironment
USPIO	Ultrasmall superparamagnetic iron oxide nanoparticle
